# Characterization of *POLE* c.1373A > T p.(Tyr458Phe), causing high cancer risk

**DOI:** 10.1007/s00438-023-02000-w

**Published:** 2023-03-01

**Authors:** Mariève J. Rocque, Vilde Leipart, Ashish Kumar Singh, Pilar Mur, Maren F. Olsen, Lars F. Engebretsen, Edgar Martin-Ramos, Rosa Aligué, Pål Sætrom, Laura Valle, Finn Drabløs, Marit Otterlei, Wenche Sjursen

**Affiliations:** 1 Department of Clinical and Molecular Medicine, NTNU—Norwegian University of Science and Technology, 7030 Trondheim, Norway; 2 Department of Medical Genetics, St. Olavs Hospital, 7030 Trondheim, Norway; 3 Faculty of Environmental Sciences and Natural Resource Management, Norwegian University of Life Sciences, NMBU, 1432 Ås, Norway; 4 Hereditary Cancer Program, Catalan Institute of Oncology, Oncobell Program, Bellvitge Biomedical Research Institute (IDIBELL), Hospitalet de Llobregat, Barcelona, Spain; 5 Centro de Investigación Biomédica en Red de Cáncer (CIBERONC), Madrid, Spain; 6 Department of Biomedical Sciences, School of Medicine, University of Barcelona, IDIBAPS, Barcelona, Spain; 7 Department of Computer and Information Science, NTNU—Norwegian University of Science and Technology, 7491 Trondheim, Norway; 8 Bioinformatics Core Facility-BioCore, NTNU—Norwegian University of Science and Technology, 7491 Trondheim, Norway; 9 K.G. Jebsen Center for Genetic Epidemiology, NTNU—Norwegian University of Science and Technology, 7030 Trondheim, Norway

**Keywords:** Proofreading polymerase-associated polyposis, PPAP, Mutator phenotype, Protein modeling, Variant classification

## Abstract

The cancer syndrome polymerase proofreading-associated polyposis results from germline mutations in the *POLE* and *POLD1* genes. Mutations in the exonuclease domain of these genes are associated with hyper- and ultra-mutated tumors with a predominance of base substitutions resulting from faulty proofreading during DNA replication. When a new variant is identified by gene testing of *POLE* and *POLD1*, it is important to verify whether the variant is associated with PPAP or not, to guide genetic counseling of mutation carriers. In 2015, we reported the likely pathogenic (class 4) germline *POLE* c.1373A > T p.(Tyr458Phe) variant and we have now characterized this variant to verify that it is a class 5 pathogenic variant. For this purpose, we investigated (1) mutator phenotype in tumors from two carriers, (2) mutation frequency in cell-based mutagenesis assays, and (3) structural consequences based on protein modeling. Whole-exome sequencing of two tumors identified an ultra-mutator phenotype with a predominance of base substitutions, the majority of which are C > T. A SupF mutagenesis assay revealed increased mutation frequency in cells overexpressing the variant of interest as well as in isogenic cells encoding the variant. Moreover, exonuclease repair yeast-based assay supported defect in proofreading activity. Lastly, we present a homology model of human POLE to demonstrate structural consequences leading to pathogenic impact of the p.(Tyr458Phe) mutation. The three lines of evidence, taken together with updated co-segregation and previously published data, allow the germline variant *POLE* c.1373A > T p.(Tyr458Phe) to be reclassified as a class 5 variant. That means the variant is associated with PPAP.

**Supplementary Information:**

The online version contains supplementary material available at 10.1007/s00438-023-02000-w.

## Introduction

Germline pathogenic variants affecting the proofreading activity of *POLE* (OMIM *174,762) and *POLD1* (OMIM *174,761) cause a dominantly inherited colorectal cancer (CRC) predisposing syndrome called polymerase proofreading-associated polyposis (PPAP) (OMIM 615083 and 612591) (Briggs and Tomlinson 2013; Rayner et al. 2016). *POLE* and *POLD1* encode the catalytic subunit of the two main replicative polymerases in eukaryotes: polymerase epsilon and polymerase delta, respectively, and cancer-associated mutations affect their proofreading or intrinsic DNA repair activity. PPAP is a high-penetrance cancer syndrome characterized by the presence of multiple colorectal adenomas and carcinomas, and increased risk to extracolonic tumors including endometrial, ovarian, brain, breast and pancreatic cancers, and tumors, benign and malignant, of the upper gastrointestinal tract, among others (Palles et al. 2013; Rohlin et al. 2014; Hansen et al. 2015; Spier et al. 2015; Castellsague et al. 2019; Vande Perre et al. 2019; Mur et al. 2020; Siraj et al. 2020). Similarly, somatic proofreading *POLE* and *POLD1* mutations occur in colorectal and endometrial cancers, and to a lesser extent, in other tumor types (see (Rayner et al. 2016)). When a new variant is identified by gene testing of *POLE* and *POLD1,* it is important to verify whether the variant is associated with PPAP or not, to guide the genetic counseling of patients and family members harboring the variant.

In 2015, we reported the presence of *POLE* (NM_006231.3) c.1373A > T p.(Tyr458Phe) in a large Norwegian family with high incidence of colorectal adenomas and carcinomas as well as malignant tumors of the pancreas, ovaries, and small intestine (Hansen et al. 2015). This variant, like the other cancer-associated pathogenic *POLE* variants, is located in the exonuclease domain, which determines the proofreading activity of the polymerase. More specifically, it affects a highly conserved residue in the Exo III motif. As indicated in our original report, variants located at the corresponding residue in orthologues significantly decrease exonuclease activity (Derbyshire et al. 1991; Soengas et al. 1992; Abdus Sattar et al. 1996; Kühn and Knopf 1996; Hansen et al. 2015). A more recent study showed that somatic variants affecting the same residue have been identified in colorectal (p.Tyr458His; p.Tyr458Cys) and brain (p.Tyr458His) hyper- and ultra-mutated cancers [13.5–753.2 mutations per Mb (mut/Mb)], suggesting a pathogenic nature, although not confirmed due to lack of mutational signature information (Campbell et al. 2017).

Tumors with *POLE* exonuclease mutations, either germline or somatic, accumulate base substitutions as consequence of the proofreading deficiency. They are considered hypermutated when they harbor > 10 mutations/Mb, or ultra-mutated when they harbor > 100 mutations/Mb. The accumulated changes correspond to COSMIC mutational signature SBS10 (Alexandrov et al. 2013), which recently was divided into SBS10a and SBS10b. The former demonstrates a bias towards *C* > *A* in a TCT context while the latter demonstrates a bias towards *C* > *T* in a TCG context (Alexandrov, Kim et al. 2020). Proofreading deficient tumors are generally microsatellite stable, however, microsatellite instable (MSI) tumors have also been reported (Elsayed et al. 2015; Mur et al. 2020). In this context, COSMIC mutational signature SBS14 has been associated with combined *POLE* exonuclease domain mutation and mismatch repair (MMR) deficiency, and SBS20, with *POLD1* and MMR deficiency or MSI (Alexandrov et al. 2013; Alexandrov et al. 2020).

Here, we aim to provide functional evidence, including tumor mutational burden and spectrum, functional assays and in silico structural analyses, together with updated segregation data that support pathogenicity for *POLE* c.1373A > T p.(Tyr458Phe).

## Materials and methods

### Carrier family

**Fig. 1:**
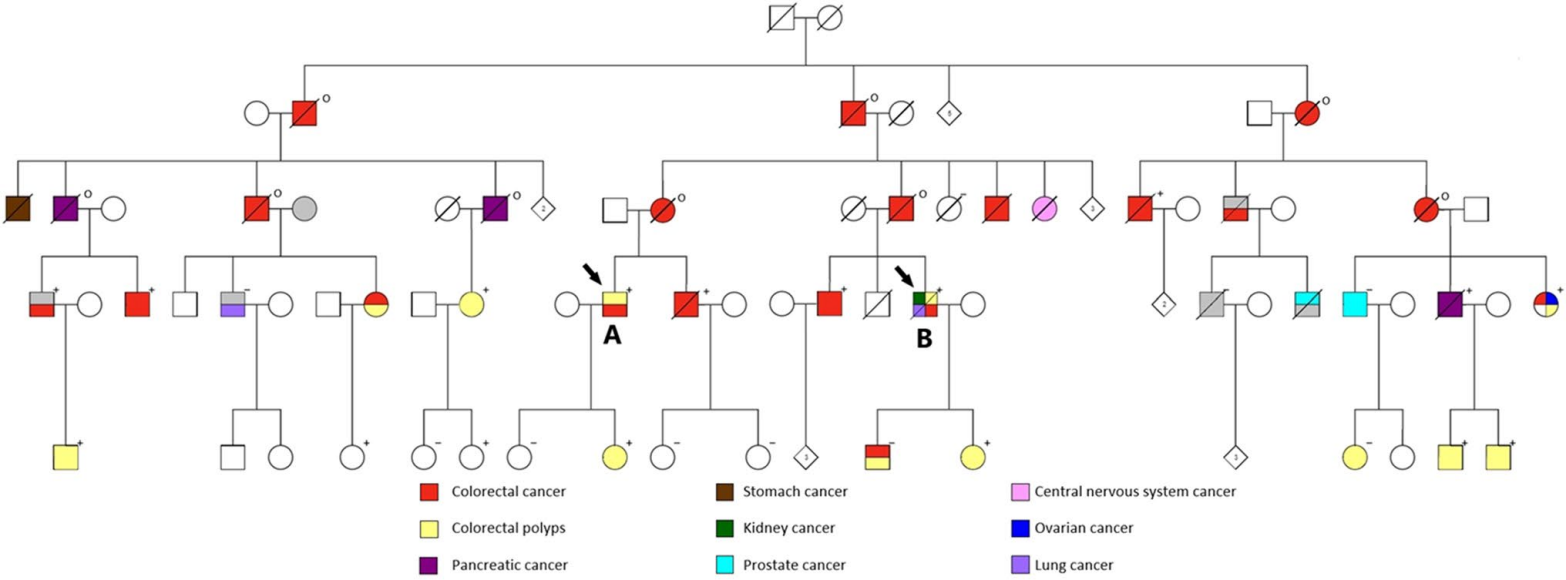
Pedigree of the carrier family. Demonstrating 24 informative meiosis (affected mutation carriers) in addition to index patient (**B**). Family members tested for mutations in the POLE gene are marked with either a plus (+) or a minus (-) sign where (+) represents detection of POLE c.1373A > T mutation (n = 16) and (-) represents wild-type of POLE. A small o is marked next to family members who are obligate carriers (n = 9). An arrow points to the two patients (**A** and **B**) whose tumors were sequenced in the present study

The clinical characteristics of the family carrying *POLE* c.1373A > T p.(Tyr458Phe) are updated in the extended pedigree (Fig. 1). In the previous paper, we reported two families with this *POLE* variant (Hansen et al. 2015). However, they have been found to belong to the same family. The four generations of the family from whom we have information of cancer phenotype, comprise 27 mutation carriers, including 10 obligate carriers. Of the 27 mutation carriers, 25 have been diagnosed with cancer; CRC (*n* = 16) or pancreatic cancer (*n* = 3) with debut age range: 38–63 years old, and/or multiple adenomas (only adenomas: *n* = 6). Three of the mutation carriers affected with CRC also developed other cancer types (cancer of small intestine, bilateral ovarian cancer, kidney cancer, and lung cancer). The two healthy mutation carriers are both in their 20s.

Ten family members have tested negative for the *POLE* variant, whereof five without any cancers or adenomas. Four non-mutation carriers have developed cancer, however, none of these (cancer in prostate, lung, bladder and one neuroendocrine tumor in colon) are cancer types associated with PPAP. The tenth non-mutation carrier had one adenoma in her 40s.

### Tumor analysis

The regional committees for medical and health research ethics has approved the project (REK#14805). Written informed consent was obtained from the mutation carriers whose tumors were analyzed with whole-exome sequencing (WES).

Tumors’ MMR deficiency was assessed by MSI analysis and immunohistochemistry of MMR proteins at St. Olavs Hospital’s Pathology Department, as previously described (Sjursen et al. 2009).

DNA extraction from formalin-fixed paraffin-embedded (FFPE) tumors was performed using the QIAamp DNA FFPE Tissue Kit (Qiagen) and QIAcube robot. Genomic DNA was extracted from matched blood using DSP DNA Midi Kit (Qiagen) and the QIASymphony robot.

WES of paired blood and tumor DNA was carried out at Centro Nacional de Análisis Genómico (Barcelona, Spain). Briefly, exomes were captured by Agilent v.5 and sequenced at an average depth of 150 × on a Hi-Seq2000 (Illumina). FASTQ files were obtained and bioinformatic analyses were performed. This included alignment of raw reads to human reference genome hg19 with BWA (option-mem), and somatic variant calling the aligned reads with GATK MuTect2. Quality control was done using FASTQC and GATK toolkits; only variants that passed quality control (“PASS” in the VCF files’ “FILTER” column) were kept for subsequent analyses.

Mutalisk web-based toolkit (Lee et al. 2018) was used for somatic mutation analysis. VCF files were uploaded to the web-based portal and signature decomposition was conducted by linear regression using “30 currently known standard signatures” without specifying the cancer type. The data were also analyzed with the AlexandrovLab/SigProfilerExtractor (Islam and Díaz-Gay 2022).

### SupF mutagenesis assay

The supF mutagenesis assay was carried out in the HEK293T cell line, with slight modifications to a previously reported protocol (Lin et al. 2011; Ræder et al. 2019) in cells overexpressing *POLE* and in an isogenic cell line harboring *POLE* c.1373A > T.

The HEK293T cell line encoding the POLE c.1373A > T variant (HEK293T^p.Tyr458Phe/+^) was generated using CRISPR/Cas9 technology as described in (Ran et al. 2013). Briefly, single guide RNAs (sgRNAs) were selected using the online CRISPR Design Tool (http://tools.genome-engineering.org) (Cong, Ran et al. 2013), cloned into pSpCas9(BB)-2A-GFP (PX458, a kind gift from Dr. Feng Zhang, Addgene plasmid #48138) and sequence verified using the SpCas9:sgRNA vector plasmid (Table S1). HEK293T cells were transfected with the plasmid containing the sgRNA as well as a manually designed single-stranded oligonucleotide serving as repair template for precise mutagenesis (Table S1). Silent mutations were incorporated in the repair template to prevent re-cleavage at the site of interest. These silent mutations also served to introduce a BfaI restriction site, which was used to screen clones for positive gene editing. The Amaxa® Cell Line Nucleofector® Kit V (Lonza) was used for transfection. After 48 h, transfected cells were sorted by fluorescence-activated cell sorting. Four days later, clonal cell lines were isolated by limiting dilution. After clonal expansion, gDNA was isolated using the DNeasy Blood & Tissue kit (QIAGEN; Redwood City, CA, USA). A fragment of DNA incorporating the site of interest and silent mutation was amplified by PCR using the POLE c.1373 primers listed in Table S1. PCR products were digested with BfaI (NEB) for 1 h at 37 °C. Digestion reactions were visualized on a 2% metaphor agarose gel. The HEK293T CRISPR control cell line (HEK293T^control^) was generated as described above except that PX458 was transfected without sgRNA insert to ensure no targeted cutting. Gene editing was validated by Sanger sequencing of PCR products using the BigDye Terminator v3.1 Cycle Sequencing Kit (Life Technologies). Potential off-target sites were evaluated by the T7E1 mismatch cleavage assay and absence of off-target modification was confirmed by Sanger sequencing.

The supF shuttle vector pSP189 was transfected (X-tremeGENE HP transfection reagent, Roche Diagnostics) into the isogenic HEK293T cells, where it was replicated by the mammalian replication machinery. Isogenic cells encoding *POLE* c.1373A > T were generated by CRISPR/Cas9 (see Online Resource Supplementary Table S1). In the overexpression system, HEK293T cells were co-transfected (X-tremeGENE HP transfection reagent) with the reporter plasmid and one of the different pEF6-POLE-EYFP plasmids: wild-type (WT); *POLE* c.1373A > T p.(Tyr458Phe); *POLE* c.1270C > G p.(Leu424Val) (positive control); and c.1089C > A p.(Asn363Lys) (positive control). The reporter plasmids were isolated from the cells (Wizard® Plus SV miniprep kit, Promega) at 22 h post-transfection in the overexpression system and 96 h post-transfection in the isogenic cell lines. Recovered plasmids were digested with DpnI (NEB) for 4 h at 37 °C and purified (ethanol precipitation). Recovered plasmids were transformed in MBM7070 bacteria, which were grown on LB-agar plates containing 100 µg/ml 5-bromo-4-chloro-3-indolyl-β-D-galactopyranoside, 100 µg/ml ampicillin and 2 µM isopropylthio-β-galactoside. Mutation frequency was measured by counting blue (representing WT supF gene) and white (representing a mutated supF gene) colonies. Recovered plasmid DNA from each biological replica was transformed until obtaining 8,000–10,000 colonies per replica. One-tailed paired statistical *t* tests were used to assess the differences between WT and variants.

### Exonuclease repair yeast-based assay

The exonuclease repair ability of POLE in the presence of missense exonuclease domain variants was tested in a yeast assay. The pFA6α-KanMX6 vector was used to clone WT pol2 gene from *Schizosaccharomyces pombe* (yeast homolog of human POLE), as previously described (Esteban-Jurado et al. 2017). Human POLE variants p.(Tyr458Phe), and known pathogenic variants p.(Asn363Lys), and p.(Leu424Val), were generated by the QuikChange II XL Site-Directed Mutagenesis Kit (Stratagene, La Jolla, CA), according to manufacturer’s conditions. Sanger sequencing was used to verify the presence of the variants. Primer sequences are detailed in Mur et al. (2020) and Online Resource Supplementary Table S2.

Adenine-defective (ade6-485) *S. pombe* was transfected with the linearized plasmid carrying pol2 WT and variants, grown in adenine-deficient media. Experiments were performed as described (Mur et al. 2020) and calculations of mutation rates to compare ade6-485 allele reversion rate of the pol2 WT (negative control) with the corresponding variants were carried out using Mann–Whitney nonparametric test*.*

### Protein homology structural modeling

The presented homology model of human DNA POL ε catalytic subunit A (UniProt ID: Q07864) was based on the crystal structure of yeast DNA polymerase epsilon (*Saccharomyces cerevisiae*) (PDB-ID: 4PTF) (Jain and Rajashankar 2014). Detailed methodology is included in Online Resource Supplementary Fig. S1.

The homology model was also used to confirm the position of Tyr458 within the DNA binding cleft. The only structure identified with a DNA fragment near Tyr458 is that of *Pyrococcus abyssi* DNA polymerase 1 (UniProt ID: P0CL76 and P0CL77). To find the homologous residues in PDB ID: 4FLU, the amino acid sequence of *P. abyssi* DNA polymerase 1 was aligned with the amino acid sequence of human Pol ε catalytic subunit A (UniProt ID: Q07864) using Clustal Omega.

### Interpretation of pathogenicity

The revised ACMG guidelines to classify POLE/D1 exonuclease domain variants as recommended by Mur et al. (2020), and the CanVIG-UK Consensus Specification for Cancer Susceptibility genes of ACGS guidelines version 2.12 (Garrett et al. 2021), were followed. According to revised guidelines, the ACMG criteria may be used at different levels out from the strengths behind the category evidences for pathogenicity (or for benign) (Richards et al. 2015), compared to the original paper from 2015. For example, the PP4 supporting evidence against pathogenicity may be used at moderate or strong level, out from the tumors hyper-mutability and type of Cosmic signature (Mur et al. 2020). The CanVIG guidelines refer to Jarvik and Browning (2016), for grading of the supporting segregation category PP1. Their formula calculates the probability (*N*) that the observed variant-affected status data occur by chance, rather than due to co-segregation, and the probability *N* = (1/2)^*m*^, where m is the number of meioses of the variant of interest that are informative for co-segregation. PP1 can be used as a strong evidence when *N* ≤ 1/32 (> 5 informative meioses) if in one family. Mur et al. (Mur et al. 2020) suggested using PP3 (multiple lines of computational evidence support deleterious effect) when the meta-predictor REVEL (Ioannidis et al. 2016) score was ≥ 0.35. We also used the CADD (Rentzsch et al. 2019) score to look into the PP3 evidence.

## Results

### Tumor MMR status, mutation burden and mutational signatures

WES was performed in two CRCs obtained from two members of the carrier family: patient A (Fig. 1, corresponds to IV:21 in Hansen et al. (2015)) and patient B (Fig. 1, corresponds to IV:17 in Hansen et al. (2015)). Patient A has removed multiple colorectal adenomas and was recently diagnosed with an MMR-deficient CRC at age 56 (MSI, loss of PMS2 and partial loss of MLH1). Patient B had polyposis from 35 year of age and was diagnosed with an MMR-deficient CRC at 42 (MSI and loss of MSH6 protein) as well as cancer in small intestine (jejunum and duodenum) at age 54 and 57, respectively. Later he has also developed kidney and lung cancer, and he is now deceased. In addition to *POLE* c.1373A > T, patient B also carried another variant in *EXO1*, c.458C > T p.(Ala153Val), whose deleterious effect remains to be elucidated. Unfortunately, no MMR-proficient tumors from other family members were obtained.

WES was successfully performed in both blood and tumor DNA, with a mean coverage depth for the sequenced FFPE DNA of 181 (95% of bases covered with > 15 reads) for patient A and 157 (93% of bases covered with > 15 reads) for patient B. After filtering out all germline variants, both tumors showed ultra-mutator phenotypes, with 449.8 and 442.3 mut/Mb, respectively (Table 1).

**Table 1:** Clinic-pathological data and results from WES for the two tumors included in this study

Patient Variants	A *POLE* c.1373A > T p.(Tyr458Phe)	B *POLE* c.1373A > T p.(Tyr458Phe) *EXO1* c.458C > T p.(Ala153Val)
Tumor type (age at diagnosis)	CRC (56)	CRC (42)
MSI status	MSI-H	MSI-H
MMR expression (immunohistochemistry)	PMS2 loss MLH1 partial loss	MSH6 loss
Mean coverage depth	181.1	156.9
% bases with coverage depth above 15	94.9	93.2
Total number of mutations (mutation rate)		
Before QC filtering	35,673 (787.5 mut/Mb)	39,760 (877.7 mut/Mb)
After QC filter	20,377 (449.8 mut/Mb)	20,037 (442.3 mut/Mb)

**Fig. 2:**
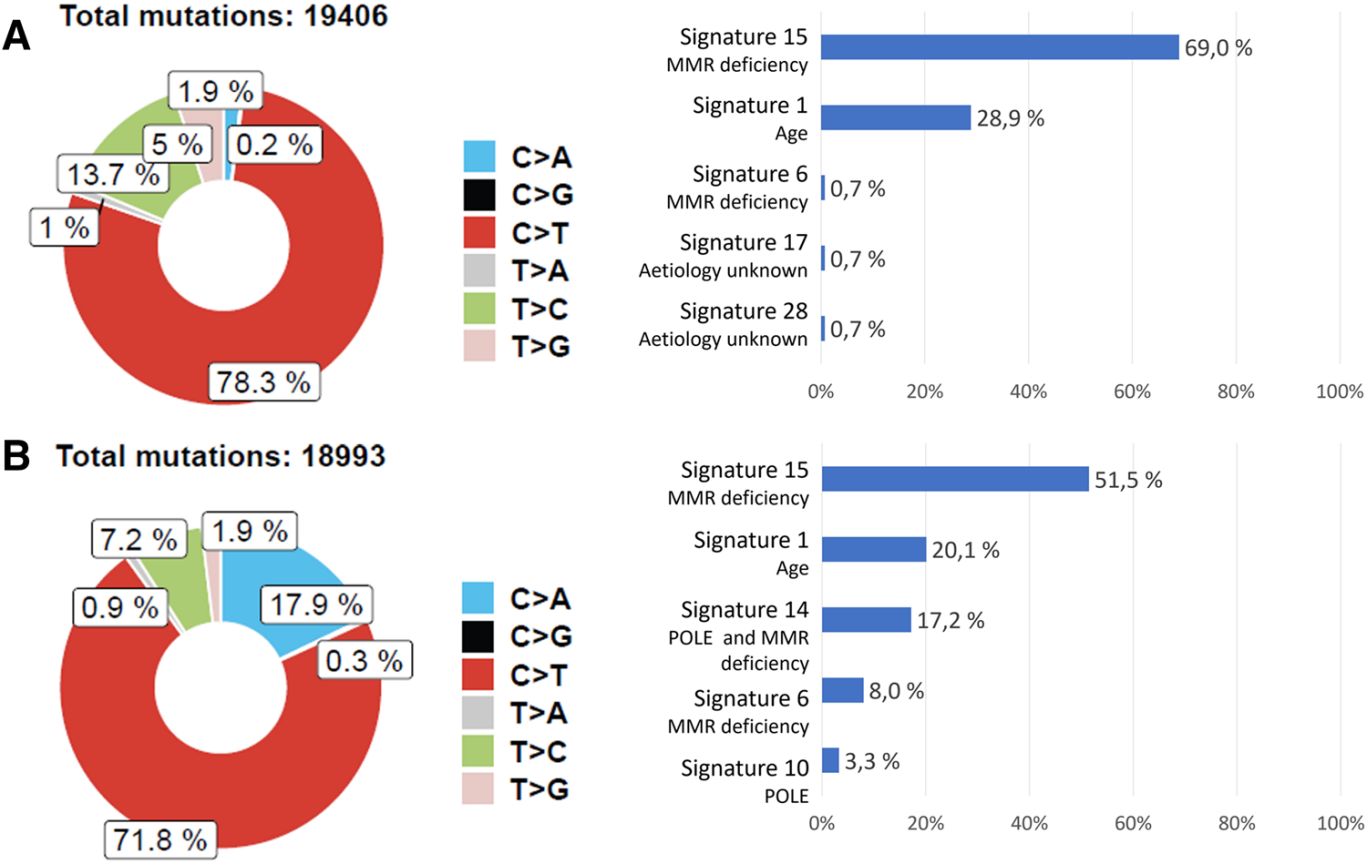
Tumor mutational spectrum and signature analysis. **a** Results for the MMR-deficient CRC from patient B (IV:17). **b** Results from the MMR-deficient CRC from patient A (IV:21). Decomposition of COSMIC mutational signatures was carried out with Mutalisk (Lee et al. 2018). Figure modified from the one generated by Mutalisk

In both tumors, most mutations leading to the ultra-mutator phenotype were base substitutions. After QC filtering patient A had a total of 19,406 base substitutions and 971 indels and patient B had a total of 18,993 base substitutions and 1044 indels, and in both tumors most base substitutions were *C* > *T* (Fig. 2).

Systematic decomposition of COSMIC mutational signatures using Mutalisk web service program (Lee et al. 2018) revealed that SBS15, associated with MMR deficiency, was the most frequent signature in both tumors, contributing to 69% (patient A) and 51.5% (patient B) of the identified signatures. Proofreading defective-associated signatures were only identified in the tumor of patient B, including SBS10 (3.3% of the identified signatures), and SBS14 (17.2% of the identified signatures) (Fig. 2; Online Resource Supplementary Fig. S2). The AlexandrovLab/SigProfilerExtractor gave comparable results to the Mutalisk results in that only one of the tumors showed POLE-related signature 14. The canonical POLE-related mutations (*T*[*C* > *A*]*T* and *T*[*C* > *T*]*G*) constitute 20% and 30%, and 6% and 5% for tumor B and A, respectively, of all *C* > *A* and *C* > *T* mutations (data not shown).

### Increased mutation frequency detected by supF mutagenesis assay in HEK293T

To examine the impact of POLE p.(Tyr458Phe) on the proofreading activity of the polymerase, we performed a supF mutagenesis assay, comparing the variant’s effect to that observed in WT controls and known pathogenic variants c.1270C > G p.(Leu424Val) (Palles et al. 2013; Barbari et al. 2018; Castellsague et al. 2019; Hamzaoui et al. 2020; Mur et al. 2020) and c.1089C > A p.(Asn363Lys) (Rohlin et al. 2014; Hamzaoui et al. 2020). HEK293T cells were selected as model cell line due to their mismatch repair deficiency (promoter silencing in *MLH1* (Trojan et al. 2002)), thereby allowing for a genetically sensitized background. Two model systems using the supF assay were developed: (1) one model where *POLE* variants were overexpressed; (2) and the other model, where *POLE* c.1373A > T p.(Tyr458Phe) was introduced in the isogenic HEK293T cell line via CRISPR/Cas9.

**Fig. 3:**
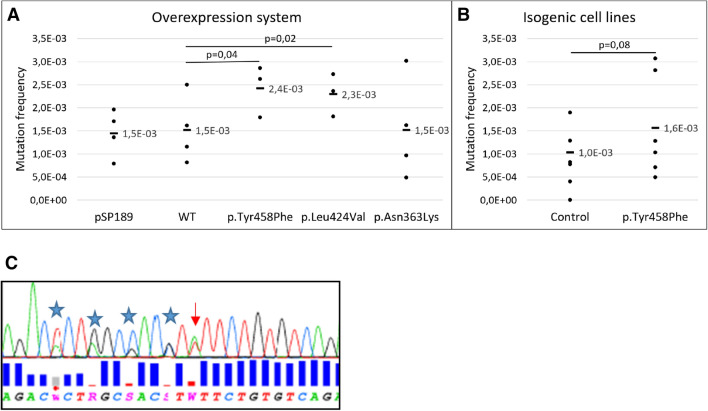
SupF assay results. **a** Mutation frequency obtained by overexpressing POLE-EYFP in HEK293T cells. Overexpression vectors include POLE WT, *POLE* c.1373A > T p.(Tyr458Phe), and positive controls c.1270C > G p.(Leu424Val) and c.1089C > A p.(Asn363Lys). Three or four biological replicates were performed (*dots*) per variant, and the average mutation frequency for each sample (*black horizontal bars*) was calculated. Considering all replicates, the total number of colonies counted were: reporter plasmid (pSP189) only (*n* = 40,030), WT (*n* = 38,953), c.1373A > T (*n* = 35,733), c.1270C > G (*n* = 30,327), and c.1089C > A (*n* = 31,019). **b** Mutation frequency obtained in isogenic HEK293T cells. Six biological replicates were performed (*dots*) per variant and the average mutation frequency for each sample (*black horizontal bars*) was calculated. The total number of colonies counted were: HEK293Tcontrol (*n* = 48,436) and HEK293Tc.1373A > T/ + (*n* = 53,903). Given p is from one-tailed paired *t* tests. **c** Sanger sequencing of DNA from isogenic HEK293T cells showing the *POLE* c.1373A > T (*arrow*) in addition to silent mutations (*stars*) introduced in order to avoid the edited cells to be recognized by sgRNA and cut by Cas9

In the overexpression model, the reporter plasmid was isolated from the HEK293T cells after 22 h. This replication window was selected due to viability problems in cells overexpressing POLE-EYPF over time (Online Resource Supplementary Fig. S3). Despite the short replication window, overexpression of *POLE* c.1373A > T p.(Tyr458Phe) and c.1270C > G p.(Leu424Val) led to a statistically significant increase in mutation frequency compared to the WT (Fig. 3a), *p* = 0.04 and *p* = 0.02, respectively. On the other hand, *POLE* c.1089C > A p.(Asn363Lys), with high inter-experiment variability, did not show statistically significant differences compared to WT.

The spontaneous mutation rates of genetically modified HEK293T (heterozygote for *POLE* c.1373A > T, HEK293T^c.1373A>T/+^), generated with CRISPR/Cas9, and the corresponding control (HEK293T^control^) were tested by harvesting the reporter plasmid 96 h post-transfection. The mutation frequency was increased in HEK293T^c.1373A>T/+^ compared to the HEK293T^control^ cells (Fig. 3b). Although non-significant (*p* = 0.08) for that time frame, the observed differences further supports the mutagenic role of *POLE* c.1373A > T p.(Tyr458Phe). Figure 3c shows Sanger sequencing of DNA from isogenic HEK293T cells showing the *POLE* c.1373A > T in addition to silent mutations incorporated in the repair template to prevent re-cleavage at the site of interest.

### Defective proofreading by a yeast-based assay

**Fig. 4:**
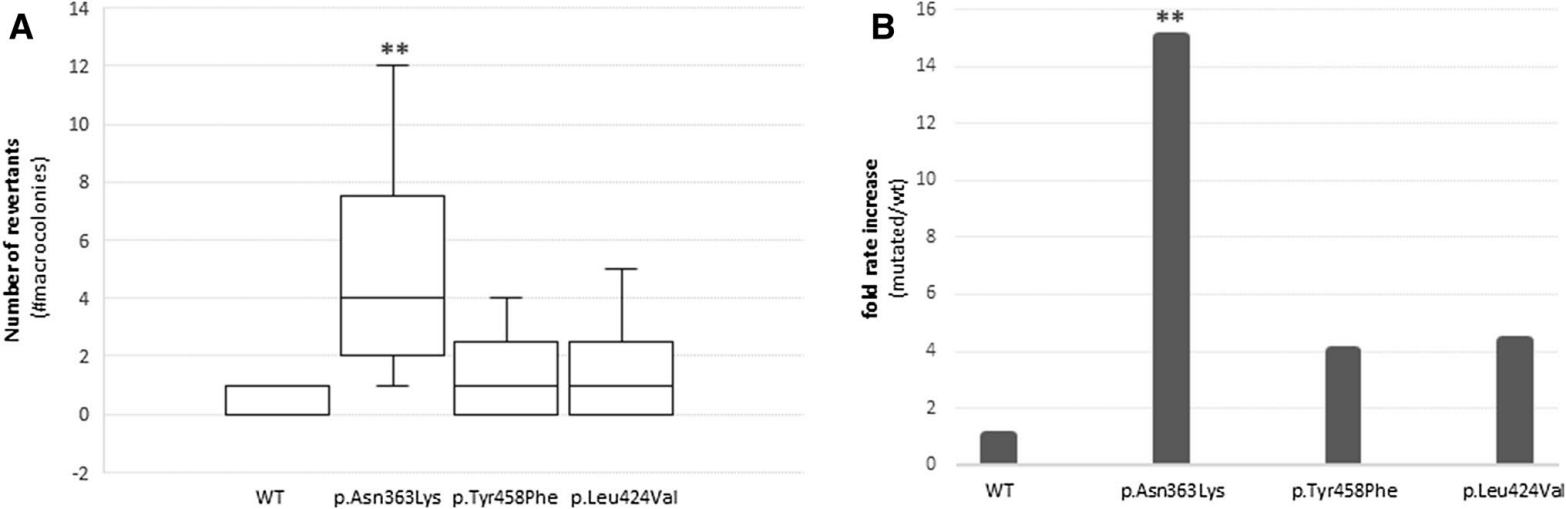
Exonuclease repair yeast-based assay**. a** Box plots showing the mutation rates of ade6-485 *S. pombe* (number of macro-colonies) for pol2 WT (negative control), and variants p.(Tyr458Phe), p.(Leu424Val) and p.(Asn363Lys)*.* **b** Fold rate increases relative to the mean number of revertants in the WT. Data obtained from three independent experiments performed in triplicate. Differences with respect to the WT clone were calculated using the Mann–Whitney nonparametric test (**p value = 0.01–0.001)

The proofreading ability of POLE in the absence (WT) and presence of p.(Tyr458Phe), p.(Leu424Val) or p.(Asn363Lys) was tested in a yeast system, as previously described (Mur et al. 2020). Results from three independent experiments performed in triplicate showed the number of revertant colonies was higher for all variants compared with the WT (fold change increase of 2.7 to 15). The differences observed were significant for p.(Asn363Lys) (positive control) (*p* = 0.00262), and non-significant for p.(Leu424Val) (positive control) (*p* = 0.26272), and p.(Tyr458Phe) (*p* = 0.26272), compared with the WT (Fig. 4) not providing conclusive evidence for p.(Tyr458Phe).

### Determination of the effect of POLE p.(Tyr458Phe) by homology modeling

**Fig. 5:**
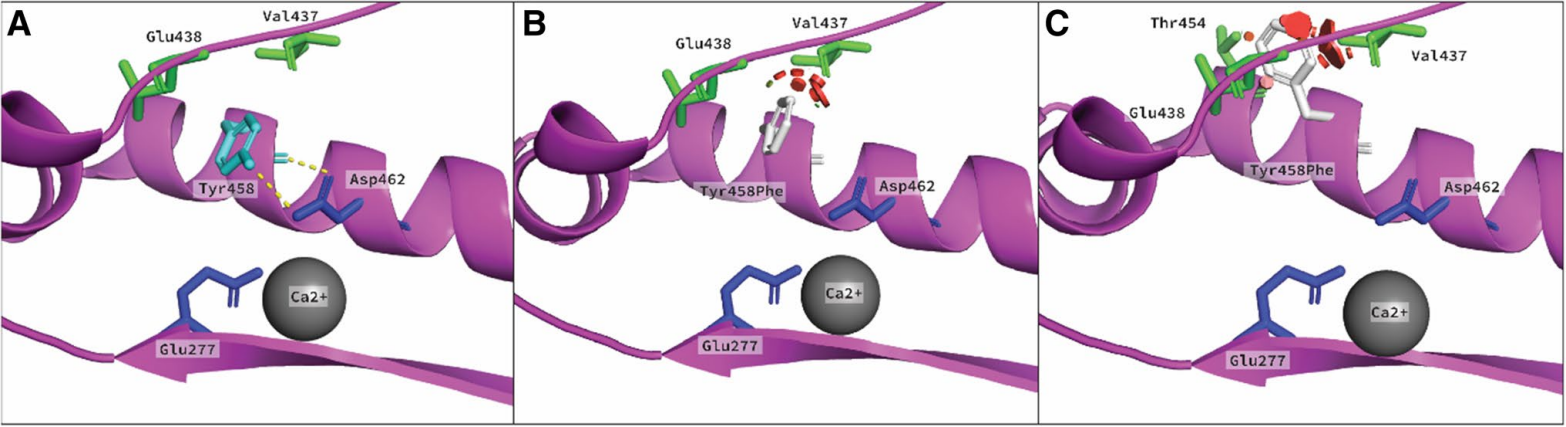
Structural changes induced by the POLE Tyr458Phe mutation**.** Tyr458 is shown as a cyan stick in the exonuclease domain (magenta). The active site residues Asp462 and Glu277 are shown as blue sticks, and the Ca^2+^-ion is shown in grey. **a** The hydrogen bond between OH-group of Tyr458 and the OD2-group of Asp462 is shown as a dotted line with the distance (3.3 Å). **b** Mutation to Phe (*white stick*) causes loss of hydrogen bond and steric clashes (*red plates*) to residue Val437 and Glu438 (*green sticks*) with rotamer option 1. **c** Phe rotamer option 2 is a similar scenario as for option 1, but additional steric clash (*red disks*) to Thr454 (green stick) (color figure online)

The homology protein model of human POLE (Online Resource Supplementary Fig. S1) was used to assess the structural effect of the highly conserved residue in the Exo III motif, POLE p.(Tyr458Phe). The side chain OH-group of Tyr458 forms a hydrogen bond with the O-group of active site residue Asp462 (Fig. 5a). Mutation of Tyr to Phe, which contains no OH-group, results in loss of this interaction. As active site residues often are supported by hydrogen bonds (Zheng et al. 2006), this mutation is likely damaging. In addition, the increase in size by substituting Tyr with Phe causes steric clashes with residues in the Exo V motif (Val437 and Glu438) and within its own α-helix (Thr454), depending on rotamer option of Phe (Fig. 5b and c), which may cause destabilization in the subdomain. The polarity is maintained after mutation, leaving the hydrophobic core intact. In sum, loss of the stabilizing H-bond to residue Asp462 in the exonuclease domain active site is predicted to have a pathogenic impact.

**Fig. 6:**
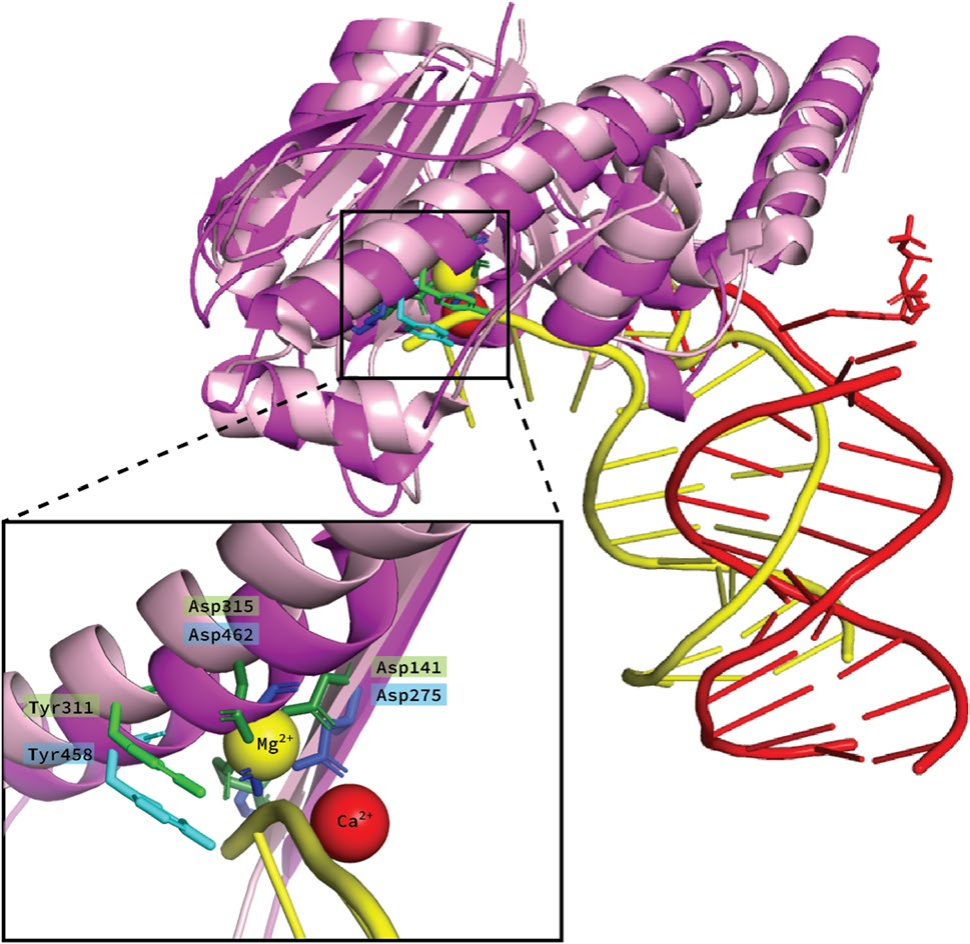
Modeling of the DNA binding cleft. The exonuclease domain of the homology model of human DNA polymerase ε catalytic subunit A (*magenta*) is shown with DNA and one Ca^2+^-ion (both in *red*), together with the aligned exonuclease domain of the *P. abyssi* DNA polymerase 1 (*light pink*, PDB ID: 4FLU (Gouge et al. 2012)), with DNA and one Mg^2+^-ion (both in *yellow*). The DNA binding pocket is highlighted in the box, where the exonuclease active site residues from both models are shown as sticks (D275, E277, D462 from Human: *blue* and D141, E143, D315 from *P. abyssi*: *green*). Tyr458 is presented as cyan stick, and the aligning and conserved Tyr311 from *P. abyssi* is shown as *green* stick. The tyrosine residues have the same rotamer orientation, and both are very close to the extended DNA strand of *P. abyssi* (*yellow*)

The human POLE homology model aligned with the exonuclease domain of *P. abyssi* confirmed that Tyr458 lies within the DNA binding cleft (Fig. 6). This additional alignment was required to depict a DNA strand near the residue of interest, Tyr458. It is clear from Fig. 6 that Tyr458 is in very close proximity to the DNA fragment, and we thereby demonstrate that Tyr458 lies within the DNA binding cleft.

### Classification of POLE c.1373A > T p.(Tyr458Phe) as pathogenic variant

Following the revised ACMG guidelines to classify *POLE/D1* exonuclease domain variants (Mur et al. 2020), and the CanVIG-UK guidelines (Garrett et al. 2021), the *POLE* c.1373A > T variant can be classified as a class 5 (pathogenic) variant (Table 2). In 2015, we reported 12 affected mutations carriers (Hansen et al. 2015), which is now extended to 25 affected mutation carriers, giving 24 informative meiosis (Fig. 1). According to the CanVIG guidelines (Jarvik and Browning 2016), the supporting segregation data evidence PP1 can be used as strong evidence because in the present family *N* = (1/2)^24^ = 1/16,777,216 (see “Materials and methods”). PP4 is used at moderate level according to Mur et al. (2020) because both of the tumors were MMR-deficient and showed ultramutation as well as one of the tumors showing the  *POLE*-associated mutational signature 14.

**Table 2:** Summary of *POLE* c.1373A > T p.(Tyr458Phe) classification according to modifications suggested in Mur et al. (2020) for the application of the ACMG/AMP guidelines specific for *POLE/D1* exonuclease domain variants and CanVIG-UK Consensus Specification for Cancer Susceptibility genes (Garrett et al. 2021)

Level and weight of evidence	Findings
PS3_ moderate	The supF mutagenesis assay supports a mutation frequency at the same order as two other verified pathogenic *POLE* variants (Figs. 3 and 4) Significant reduction of the exonuclease activity has also been shown in orthologues (Derbyshire et al. 1991; Soengas et al. 1992; Abdus Sattar et al. 1996; Kühn and Knopf 1996)
PM1_moderate	The variant is at a well-conserved position in the exonuclease domain and in the DNA binding cleft (Fig. 6)
PM2_ moderate	Not found in controls; absent from gnomAD
PP1_strong	24 informative meiosis (Fig. 1)
PP3_supportive	REVEL score: 0.49 CADD score: 24.4
PP4_moderate	Two hypermutated tumors from different patients, one of which is associated with mutational signature 10 and 14 (Fig. 2)

The combined evidence includes one strong, four moderate and one supporting evidence for pathogenicity (Table 2). According to the ACMG/AMP guidelines, this is a class 5 pathogenic variant.

## Discussion

The present study verifies that the *POLE* c.1373A > T p.(Tyr458Phe) variant is pathogenic. This is shown by ultra-mutated tumors, functional assays, protein modeling, and segregation data.

Both tumors included in this study revealed an ultra-mutated phenotype where base substitutions predominate and a prevalence of *C* > *T* base substitutions is clearly detected. This is characteristic of mutator phenotypes associated with pathogenic *POLE* exonuclease domain mutations (Network 2012; Alexandrov et al. 2013; Rayner et al. 2016). Furthermore, a previous study looking into mutation burden in different tumors reported that different amino acid substitutions to residue p.Tyr458 were associated with hypermutation (Campbell et al. 2017). They reported hypermutations by substitution of residue Tyr458 to His (in adult colorectal carcinoma (342 mut/Mb) and pediatric brain tumor (85 mut/Mb)) and to Cys (in adult colon adenocarcinomas (753.2 mut/Mb and 13.5 mut/Mb)). Our data from two colorectal carcinomas with a germline substitution to Phe at Tyr458 further support that substitutions in this residue promote cancer development.

As discussed in Mur et al. (2020), interpretation of MSI tumors from *POLE* germline variant carriers is challenging, and defective DNA mismatch repair signatures are abundant in these tumors. Unfortunately, the tumors from the patients included were all MSI and no microsatellite stable tumors were available from other family members. Despite MMR deficiency in both tumors included, which was reflected in the strong contribution of signature 15, *POLE*-associated signatures (signatures 10 and 14) were identified in one of the tumors.

Considering that both the tumors included in the present study are MSI, it cannot be excluded that a synergistic effect with MMR defect is required for an ultra-mutator phenotype to be observed for the *POLE* c.1373A > T variant. The MMR deficiency in tumors included in the present study occurred somatically most probably because of defect POLE proofreading. Considering that none of the tumors with germline *POLE* mutations at residue Tyr458 included in Campbell et al. were reported to have MMR mutations (Campbell et al. 2017), we suggest that the ultra-mutator phenotype result from the germline *POLE* variant. In fact, other studies have also demonstrated that carriers of pathogenic *POLE* variants develop tumors that are rendered MMR deficient (Elsayed et al. 2015; Jansen et al. 2016). Further, other studies have shown that tumors with combined defects in POLE exonuclease and MMR activity have different degrees of associated mutation signatures (Haradhvala et al. 2018; Hodel et al. 2020).

Both the supF mutagenesis assay and, to a lesser extent, the yeast-based assay showed a tendency of increased mutation frequency caused by p.(Tyr458Phe) compared to WT. With regards to the supF mutagenesis assay, it remains a possibility that the increased mutation frequency detected was due to the use of MMR-deficient cells. When the effect of the *POLE* exonuclease double mutated allele p.(Asp275Ala/Glu277Ala) in MMR-deficient HCT116 cells was looked into in another study, MMR deficiency was reported to be necessary for the ultra-mutator phenotype associated with PPAP (Hodel et al. 2018). However, despite using MMR-deficient cells to allow for a genetic sensitized background, the mutation frequencies detected by the supF mutagenesis assay were quite low, especially considering the hyper- and ultra-mutator phenotypes associated with *POLE* variants. Factors that may explain this discrepancy include use of exogenous DNA with a small (81 bp) reporter gene and replication within a short time window (22 or 96 h depending on the model). It is difficult to make models that directly correlate with mutation acquired over the course of several decades anywhere in the human genome. Furthermore, the supF assay is biased towards surviving clones. Therefore, mutations occurring in parts of the plasmid affecting survival of its host bacteria, e.g., mutations affecting the ampicillin resistance gene, are not detected. This will lead to an underestimate of the mutation frequency. Likewise, silent mutations within the reporter plasmid are not detected. Lack of detection of an increase in mutation frequency in samples overexpressing *POLE* c.1089C > A p.(Asn363Lys), which is reported to have a mutagenic effect (Hamzaoui et al. 2020), are likely due to low sensitivity of the assay. Likewise, the yeast assay only showed significant mutation frequency differences between the WT control and one of the positive controls, p.(Asn363Lys), but not for the other positive control p.(Leu424Val), the most recurrent germline pathogenic variant, or the p.(Tyr458Phe) variant. This shows the challenges in performing functional assays in cell systems, particularly when using different organism systems, but also suggests that p.(Tyr458Phe) has a similar effect as p.(Leu424Val). Our results show that implementation of more than one functional assay using different cell systems is recommended in order to obtain more informative results. Based on the fact that one of the two functional assays did not give significant differences between the variant and the WT control, the level of strength of PS3 in the variant classification system was reduced from strong to moderate. Moreover, functional studies in orthologues have confirmed significantly reduced exonuclease activity by substituting the equivalent tyrosine in the Exo III motif with phenylalanine; p.(Tyr497Phe) in *E. coli* DNA pol I Klenow fragment polymerase (Derbyshire et al. 1991), p.(Tyr320Phe) in bacteriophage T4 DNA polymerase (Abdus Sattar et al. 1996), p.(Tyr165Phe) in bacteriophage phi 29 DNA polymerase (Soengas et al. 1992), and p.(Tyr577Phe) in herpes simplex virus DNA polymerase (Kühn and Knopf 1996). A recent functional study in *S*. *cerevisiae* showed the equivalent Tyr, pol2-p.(Tyr473Phe) to impair the exonuclease activity in several ways; including substrate binding, active site assembly across the transition state and ejection of the substrate and/or product from the exonuclease active site (Dahl et al. 2022). These studies support the functional effect of p.(Tyr458Phe) in human cells.

We present here a tertiary structure prediction of human POLE. Structural analyses of the variant p.(Tyr458Phe) indicated structural consequences resulting in pathogenic impact. Furthermore, the homology model presented herein may be used to determine which residues in POLE are located within the DNA binding cleft. We were able to confirm that Tyr458 lies in fact in the DNA binding cleft. This is of interest as location of variants within the DNA binding cleft is suggested as moderate PM1 evidence of pathogenicity (Mur et al. 2020).

The strongest evidence for pathogenicity for POLE p.(Tyr458Phe) found in this study is the segregation score. PP1 can be used as strong evidence (Fig. 1) according to CanVIG-UK guidelines in our large family. According to Mur et al. (2020), this evidence can only be used as strong evidence when ≥ 7 informative meioses are seen across ≥ 2 families. We selected to use the CanVIG-UK guidelines in this regard because of the large size of the family. Another aspect is that this large family originally was thought to be four non-related families, which was later found to have common ancestors. Such rare pathogenic variants may sometimes only be found in one family.

According to the CanVIG-UK’s last version, the PP3 (multiple lines of computational evidence support deleterious effect) should be used when the REVEL score > 0.7. However, this threshold does seem to be too strict for the *POLE*/*POLD1* genes since several of the confirmed pathogenic variants reach a lower REVEL score for these two genes (Mur et al. 2020). The *POLE* p.(Tyr458Phe) variant reached higher CADD scores than the two positive controls.

The detection of the genetic cause of the high aggregation of cancer in the family allowed a more personalized clinical management of the carrier relatives. The clinical follow-up of carrier members has prevented cancer development. In particular, the younger generations have been subjected to annual or biannual colonoscopies, which has allowed the identification and removal of colorectal adenomas. Re-classification of this variant as a class 5 pathogenic variant will aid to ensure that patients with this variant will be offered recommended guidelines for follow-up, not only for colorectal tumors, but also for other PPAP-associated tumor types (Mur et al. 2020).

## Conclusions

In the present study, three approaches have been used to substantiate the pathogenicity of the germline *POLE* c.1373A > T p.(Tyr458Phe) variant: WES of tumor DNA, reporter-based and yeast-based mutagenesis assays and in silico protein modeling based on a homology model. To our knowledge, we are the first to study tumor mutation burden of germline variant *POLE* c.1373A > T p.(Tyr458Phe) and the first to use human isogenic cell lines to study the function of germline *POLE* variants. All three approaches included in the present study support that this variant has a pathogenic impact. We can now classify the variant as a class 5 pathogenic variant causing PPAP.

## Supplementary Information

Below is the link to the electronic supplementary material.

Supplementary file1 (PDF 1527 KB)

## Data Availability

The datasets (WES of the tumors and blood samples from two patients) generated and analyzed during the current study are not publicly available due to confidentiality and ethical concerns, but are available from the corresponding author on reasonable request.
