# Supplementary material for: Characterization of POLE c.1373A > T p.(Tyr458Phe), causing high cancer risk
Source: Mol Genet Genomics. 2023 Mar 1;298(3):555–66. doi: 10.1007/s00438-023-02000-w (PMC10133059; doi:10.1007/s00438-023-02000-w)
Supplement: Supplementary file 1 — Supplementary file1 (PDF 1527 KB) [file 438_2023_2000_MOESM1_ESM.pdf]

Supplementary material for:

Characterization of *POLE* c.1373A>T p.(Tyr458Phe), causing high cancer risk

Mariève J. Rocque[1, 2], Vilde Leipart[1,3], Ashish Kumar Singh[1, 2], Pilar Mur[4, 5], Maren F. Olsen[2], Lars F. Engebretsen[2], Edgar Martin-Ramos[6], Rosa Aligué[6], Pål Sætrom[1 ,7, 8,9], Laura Valle[4, 5], Finn Drabløs[1], Marit Otterlei[1] and Wenche Sjursen[1, 2,*]

Content:

Supplementary Fig. S1-1 to S1-5: Homology protein modeling of human POLE with supporting information.

Supplementary Fig. S2: Tumor mutational signatures

Supplementary Fig. S3: Confocal microscopy time course study of POLE-EYFP fusion protein

Supplementary Table S1: Oligonucleotide sequences used for gene editing of *POLE* c.1373A>T by CRISPR/Cas9

Supplementary Table S2: Primers used for site-directed mutagenesis

Supplementary figure S1: Homology protein modeling of human POLE

S1.1. Materials and Methods

The presented homology model of human DNA POL ε catalytic subunit A (UniProt ID: Q07864) was based on the crystal structure of yeast DNA polymerase epsilon (*Saccharomyces cerevisiae*) (PDB-ID: 4PTF) (Jain, Rajashankar et al. 2014). The template was identified through BLAST with ExPDB as the target database. The homology model was interactively modeled using DeepView (Guex and Peitsch 1997). The structural realignment was done using the implemented alignment tool, with default settings (scoring matrix: PAM200, open gap penalty: 6, extended gap penalty: 4, minimum score to be similar: 1), resulting in a sequence identity of 55%. Two positions in the alignment where manually altered (Glu112 and Leu1171) so that the respective gaps are aligned to loop regions and not secondary structure elements. The alignment was inspected visually to ensure that essential residues and regions are correctly aligned. Backbone modeling, loop building, side chain building and energy minimization was preformed using implemented tools in DeepView. The quality control was performed using the Ramachandran Plot in DeepView (**Fig. S1-1A**) in addition to Verify3D (Lüthy, Bowie et al. 1992), ERRAT (Colovos and Yeates 1993), VADAR (Willard, Ranjan et al. 2003) and ProCheck (Laskowski, MacArthur et al. 1993) (**Fig. S1-1B**). These calculations resulted in some manual improvements of side chain orientations to remove non-bonded contacts to improve the quality. For example, the overall quality factor calculated by ERRAT increased from 76.50 % to 79.46 % (**Fig. S1-1C**).

For each of the variants included in the supF mutagenesis assay and the yeast-based exonuclease repair assay, namely POLE p.Tyr458Phe, p.Leu424Val (Palles, Cazier et al. 2013), and p.Asn363Lys (Rohlin, Zagoras et al. 2014), the mutations, contacts and hydrophobic regions were analyzed using several functions of PyMol (Schrodinger 2015). Two multiple sequence alignments (MSA) were computed using Clustal Omega (McWilliam, Li et al. 2013). The first MSA included DNA polymerase ε (Q07864), δ (P28340) and α (P09884) catalytic subunit of the B-family in *Homo sapiens* (see **Fig. S1-2**). The second MSA included POLE proteins in other organisms: *H. sapiens* (Q07864), *Mus musculus* (Q9WVF7), *S. cerevisiae* (P21951), *Schizosaccharomyces pombe* (P87154), *Arabidopsis thaliana* (F4HW04) and *Dictyostelium discoideum* (Q54RD4) (see Figure S1-3).

S1.2. Results

S1.2.1. Quality control of the homology model

A)

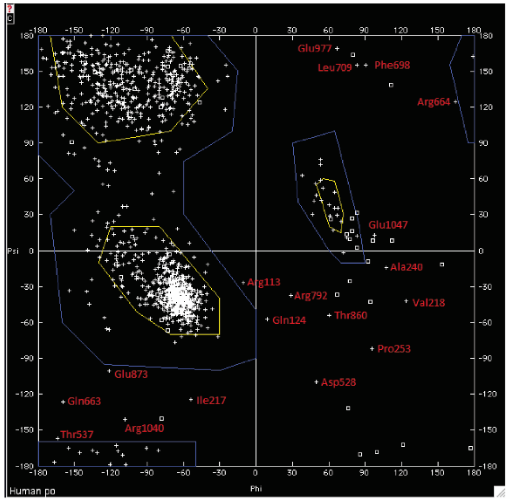

B)

Quality Scores	Template	Target
3D-1D score by Verify3D	70.86 %	67.82 %
Quality factor by ERRAT	92.45 %	79.46 %
Average 3D profile quality by VADAR	5.29	5.34
Ramachandran Plot by ProCheck		
Core	87.4 %	86.8 %
Allowed	11.6 %	11.4 %
Generous	0.4 %	1.1 %
Disallowed	0.6 %	0.7 %

C)

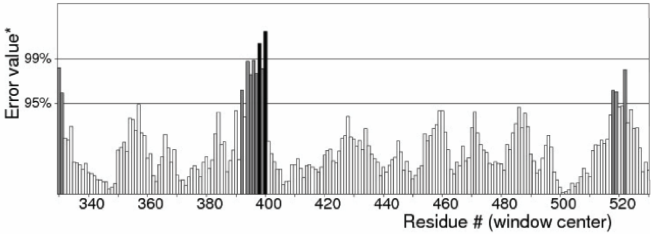

Fig. S1-1 Quality control. (A) The Ramachandran plot from DeepView. The core and allowed region are maked with the yellow line, while the generous are marked with a blue line. Outside the blue line is the dissallowed region, where all residues in this region are labeled red (+), except for glycines (▫). **(B)** The tables lists the main quality score calculated by the different programs. In general, all program uses a database of known protein structures to calculated their expected values and paramters. In this table, the score from the template is compared to the predicted target structure. The 3D-1D score assigns a structural class onto each residue, based on the location and enviroment. Presented is the number of residues with a satisfactory value. ERRAT bases the quality factor on non-bonded atom-atom interactions, and presented is the number of residues with an accepcted score. 3D profile by VADAR is calculated using the same paramters as Verify3D. The average score per residue is presented, which should be above 4 for the structue to be of satisfactory quality. Lastly, the number of residues within each region of the Ramachandran plot, calculated by ProCheck is listed. **(C)** Snapshot of the exonuclease domain in the ERRAT plot after manual improvents. The residue numbers is on the x-axis. Residues colored grey are rejected at 95 % confidence level, and residues colored black are rejected at 99 % confience level.

S1.2.2. Human homology model of POLE

The homology model includes residues 25 to 1167 (**Fig. S1-2)**, with a small missing region (residues 195 to 216). The gaps in the alignment resulted in a lower quality of the predicted structure for the N-terminal domain, while the high conservation of the exonuclease domain resulted in a high quality of this domain in the predicted structure.

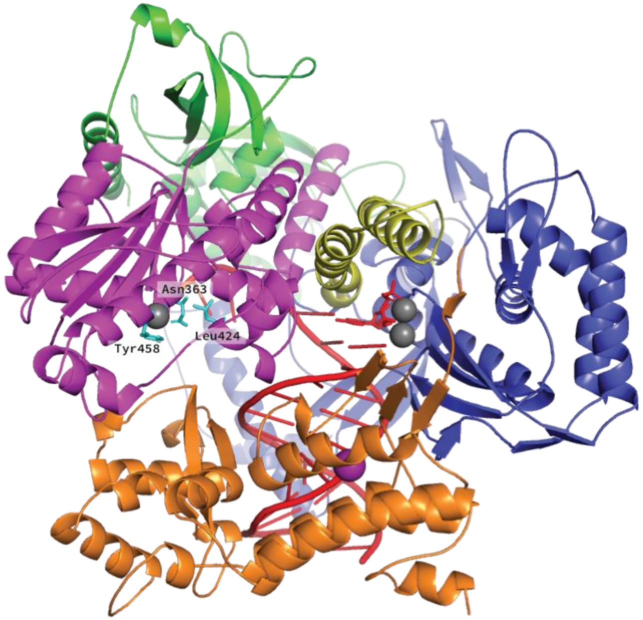

Fig. S1-2 Homology model of human DNA polymerase ε catalytic subunit A. The predicted model presented with the N-terminal domain (green), the exonuclease domain (magenta), the palm domain (blue), the finger domain (yellow) and the thumb domain (orange). Tyr458, Asn363 and Leu424 are shown as cyan sticks. The DNA, 3 dNTP (red), 1 Na^{+}-ion (purple ball) and 3 Ca^{2+}-ions (grey) are included from template structure (PDB ID: 4PTF).

S1.2.3. Functional effects in known pathogenic POLE variants is confirmed by structural analysis based on the human homology protein model

How the two confirmed pathogenic germline *POLE* mutations (*POLE* Leu424Val and Asn363Lys) affected the structure was modelled. The highly conserved Leu424 is in an α-helix in the Exo IV motif. Leu424 is not directly involved in the active site, but rather indirectly involved by maintaining the stability of the hydrophobic core (**Fig. S1-3A**) (Palles, Cazier et al. 2013). Mutation of residue 424 to valine maintains the polarity, but the decreased size reduces the interaction with the hydrophobic core and may also cause steric clashes to the neighboring amino acid, Tyr362, depending on the rotamer option of valine (**Fig. S1-3B** and **C**). Tyr 362 is highly conserved, and changes to its conformation of the side chain may cause similar destabilization for the loop-region and the two neighboring α-helixes, as seen for the Asn363Lys mutation below. In sum, the Leu424Val mutation likely results in loss of stabilizing interaction to the exonuclease domain active site -helix and is thereby predicted to have a functional impact.

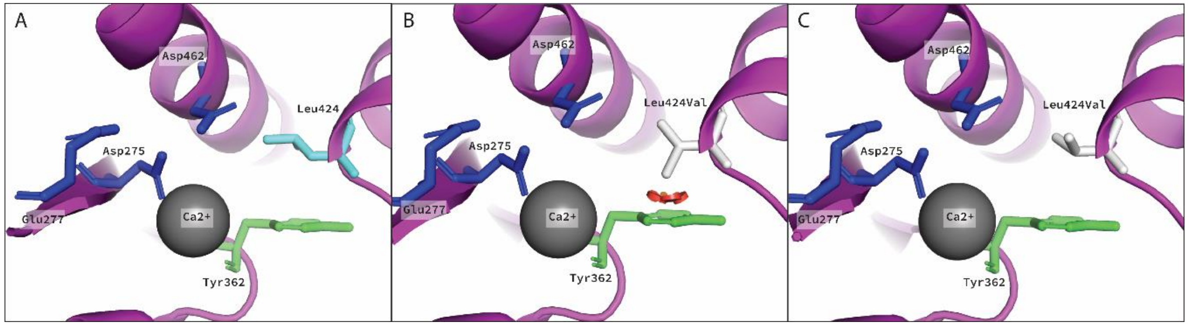

Fig. S1-3 Structural changes induced by the *POLE* Leu424Val mutation. (A) Leu424 is shown as cyan sticks in the exonuclease domain (magenta). The active site residues Asp462, Asp275 and Glu277 are shown as blue sticks, and the Ca^{2+}-ion is shown in grey. The conserved Tyr362 is shown as green sticks. (B) Mutation of residue 424 to valine (white stick) in rotamer option 1, leads to a different orientation of the side chain and causes steric clash (red plates) to Tyr362. (C) Rotamer option 2 of valine at residue 424 does not cause any steric clashes.

The highly conserved Asn363 is in a loop region upstream of one α-helix (residue 364 to 379, labelled α-helix 1 in **Fig. S1-4**), and close to a second α-helix (residue 407 to 415, labelled α-helix 2 in **Fig. S1-4**) the Exo II motif. Asn363 is not directly involved in the active site, neither are the residues in both α-helixes, but indirectly by stabilizing the opening to the active site (**Fig. S1-4A** and **Fig. S1-5**). Mutation to Lys introduces a positive charge and increases the size of the side chain. The positive charge will disrupt the strong helix dipole of α-helix 1, as residue 363 is at the carboxyl-terminal end. α-helix 2 also has a strong helix dipole with the amino-terminal end facing residue 363. The introduction of a positive charge at residue 363 will repel the positive charged end. This will lead to a destabilization of both α-helixes. The increased size will create steric clashes with α-helix 2 and its own loop region (Leu408 and Tyr362) and restrict access to the active site (**Fig. S1-4B**). Restricting access to the exonuclease domain active site resulting from substitution of Lys at Asn363 is predicted to have pathogenic effect.

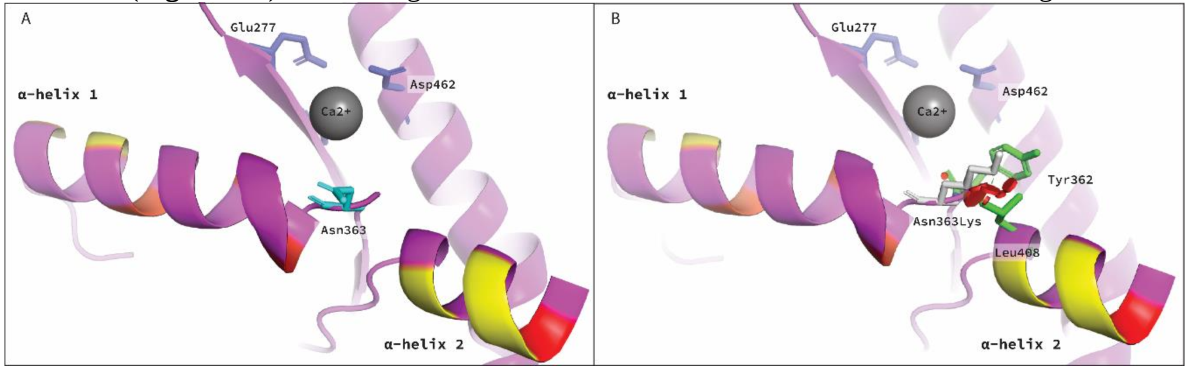

Fig. S1-4 Structural changes induced by the *POLE* Asn363Lys mutation. (A) Asn363 is shown as cyan sticks in part of the exonuclease domain (magenta). The active site residues Asp462 and Glu277 are shown as blue sticks, and the Ca^{2+}-ion is shown in grey. In α-helix 1 and 2, the positive (yellow) and negative (red) charged residues are marked. **(B)** Mutation to Lys (white stick) introduces a positive charge, destablizing the helix dipole in α-helix 1 and 2. The mutation causes steric clashes (red disks) to residue Leu408 and Tyr362 (green sticks) and restricts access to the active site.

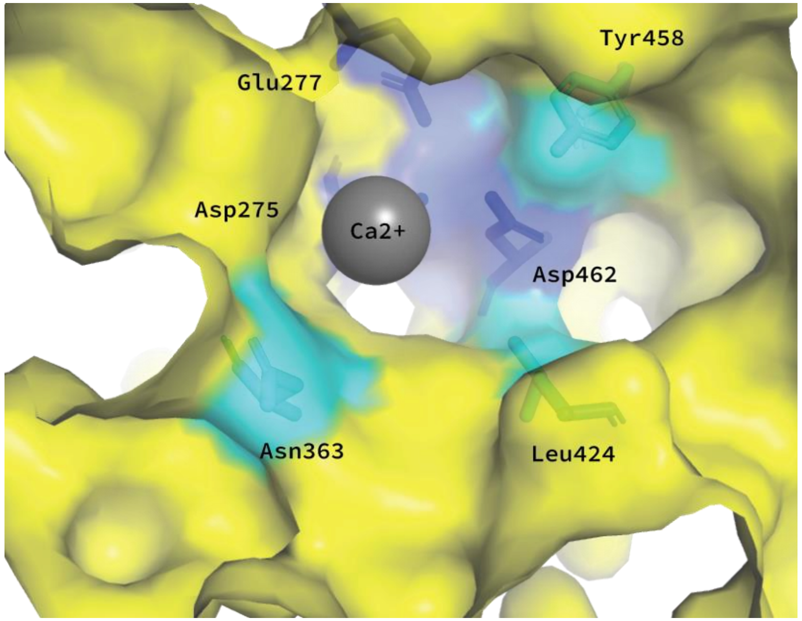

Fig. S1-5 View of the active site surface of human POLE exonuclease domain. The active site residues are colored blue and the Ca^{2+}-ion is shown in grey. Residues Tyr458, Asn363 and Leu424 are colored cyan. The surface of the active site is show in yellow, demonstaring that all the mutated residues included in the present study are in the active site.

S1.3. Conclusion

We present here a tertiary structure prediction of human POLE. Structural analyses of two POLE variants used as positive controls (Leu424Val and Asn363Lys) indicated structural consequences resulting in pathogenic impact. These results are supported by a recent study of protein stability based on X-ray crystal structure of *S. cerevisiae* DNA polymerase POL2 (pdb code: 4PTF) and double-stranded DNA from the X-ray crystal structure of *P. abyssi* B family DNA polymerase (pdb code: 4FLU). Namely, they reported a significant increase in Gibbs free energy sufficient to destabilize the protein structure when Leu 424 is substituted to Val

(Hamzaoui, Alarcon et al. 2020). Similarly, we report a loss of stabilizing interactions. Furthermore, Hamzaoui et al. (2020) demonstrate destabilization of DNA binding caused by altered DNA positioning in the exonuclease active site when Asn 363 is substituted to Lys (Hamzaoui, Alarcon et al. 2020). This corresponds well to our findings indicating that access to the exonuclease domain active site is restricted by the Asn363Lys mutation.

References Fig. S1

Colovos C and Yeates TO (1993) Verification of protein structures: patterns of nonbonded atomic interactions. Protein science : a publication of the Protein Society 2(9): 1511-1519. doi 10.1002/pro.5560020916

Guex N and Peitsch MC (1997) SWISS-MODEL and the Swiss-PdbViewer: an environment for comparative protein modeling. Electrophoresis 18(15): 2714-2723. doi 10.1002/elps.1150181505

Hamzaoui N, Alarcon F, et al. (2020) Genetic, structural, and functional characterization of POLE polymerase proofreading variants allows cancer risk prediction. Genet Med 22(9): 1533-1541. doi 10.1038/s41436-020-0828-z

Jain R, Rajashankar KR, et al. (2014) Crystal Structure of Yeast DNA Polymerase ε Catalytic Domain. PloS one 9(4): e94835. doi 10.1371/journal.pone.0094835

Laskowski RA, MacArthur MW, et al. (1993) PROCHECK: a program to check the stereochemical quality of protein structures. Journal of Applied Crystallography 26(2): 283-291. doi doi:10.1107/S0021889892009944

Lüthy R, Bowie JU, et al. (1992) Assessment of protein models with three-dimensional profiles. Nature 356(6364): 83-85. doi 10.1038/356083a0

McWilliam H, Li W, et al. (2013) Analysis Tool Web Services from the EMBL-EBI. Nucleic Acids Research 41(W1): W597-W600. doi 10.1093/nar/gkt376

Palles C, Cazier JB, et al. (2013) Germline mutations affecting the proofreading domains of POLE and POLD1 predispose to colorectal adenomas and carcinomas. Nature genetics 45(2): 136-144. doi 10.1038/ng.2503

Rohlin A, Zagoras T, et al. (2014) A mutation in POLE predisposing to a multi-tumour phenotype. International journal of oncology 45(1): 77-81. doi 10.3892/ijo.2014.2410

Schrodinger L (2015) The PyMOL Molecular Graphics System, Version 1.8.

Willard L, Ranjan A, et al. (2003) VADAR: a web server for quantitative evaluation of protein structure quality. Nucleic Acids Res 31(13): 3316-3319. doi 10.1093/nar/gkg565

Supplementary figure S2: tumor mutational signatures

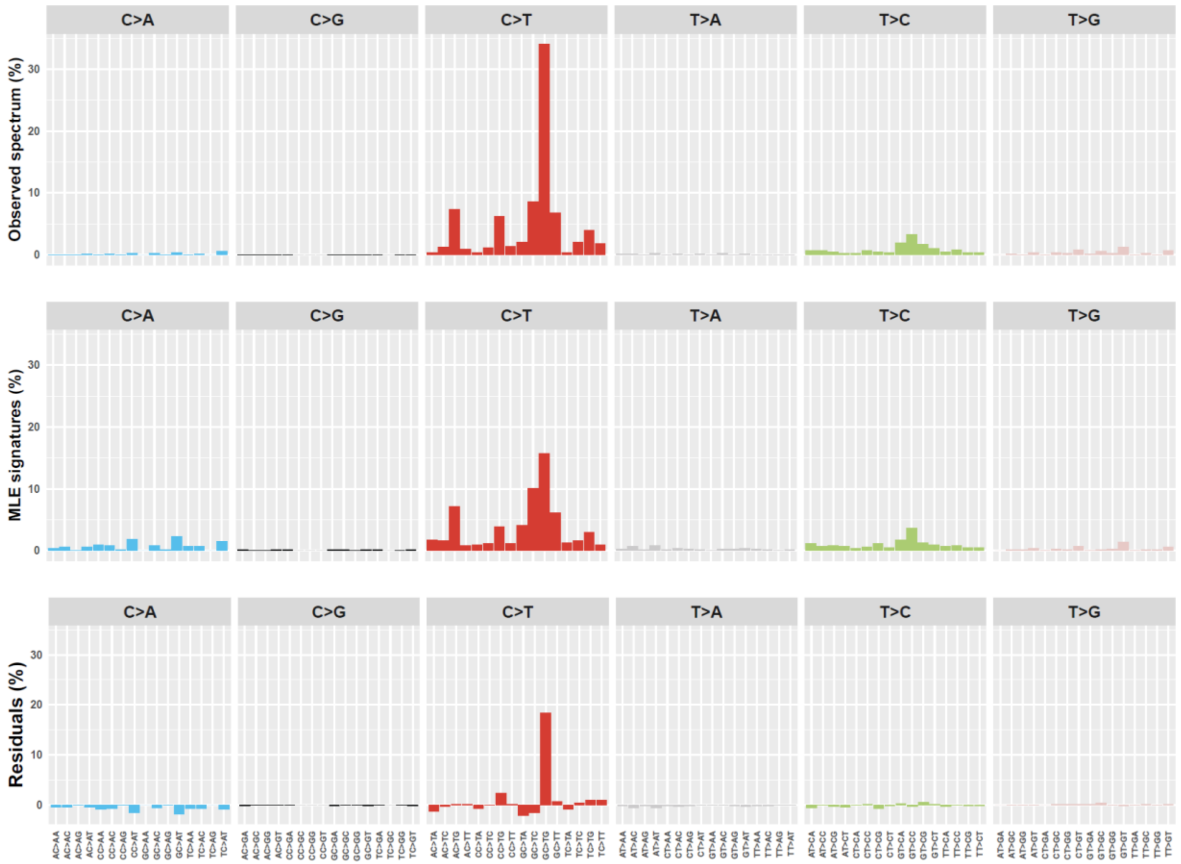

Fig. S2-1 Patient A's tumor mutational signature. The upper panel demonstrates distribution of mutations across 96 possible mutation types (6 types of substitution x 4 types of 5'base x 4 types of 3'base). The middle panel demonstrates the summation of the distribution of the decomposed signatures. The bottom panel demonstrates the difference of each base substitution subtype between the top and middle panels.

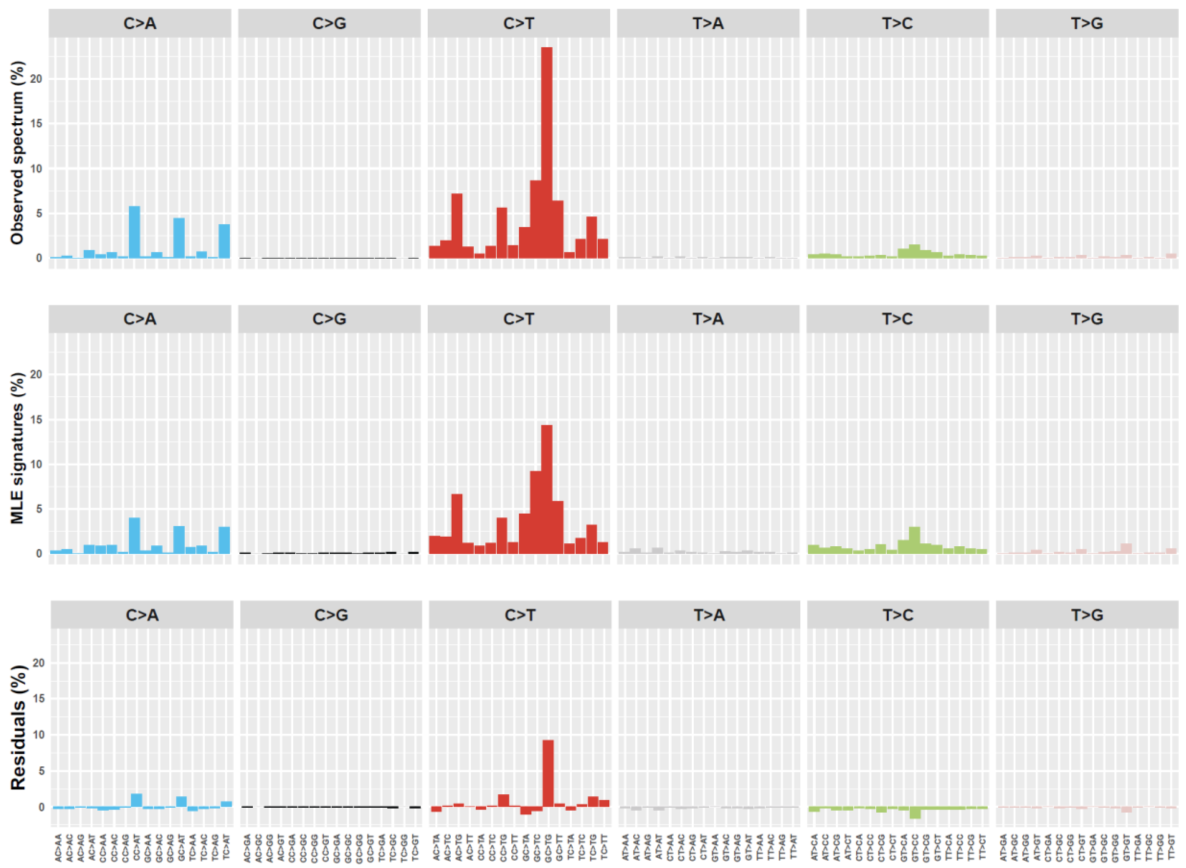

Fig. S2-2 Patient B's tumor mutational signature. The upper panel demonstrates distribution of mutations across 96 possible mutation types (6 types of substitution x 4 types of 5'base x 4 types of 3'base). The middle panel demonstrates the summation of the distribution of the decomposed signatures. The bottom panel demonstrates the difference of each base substitution subtype between the top and middle panels.

Supplementary Fig. S3:

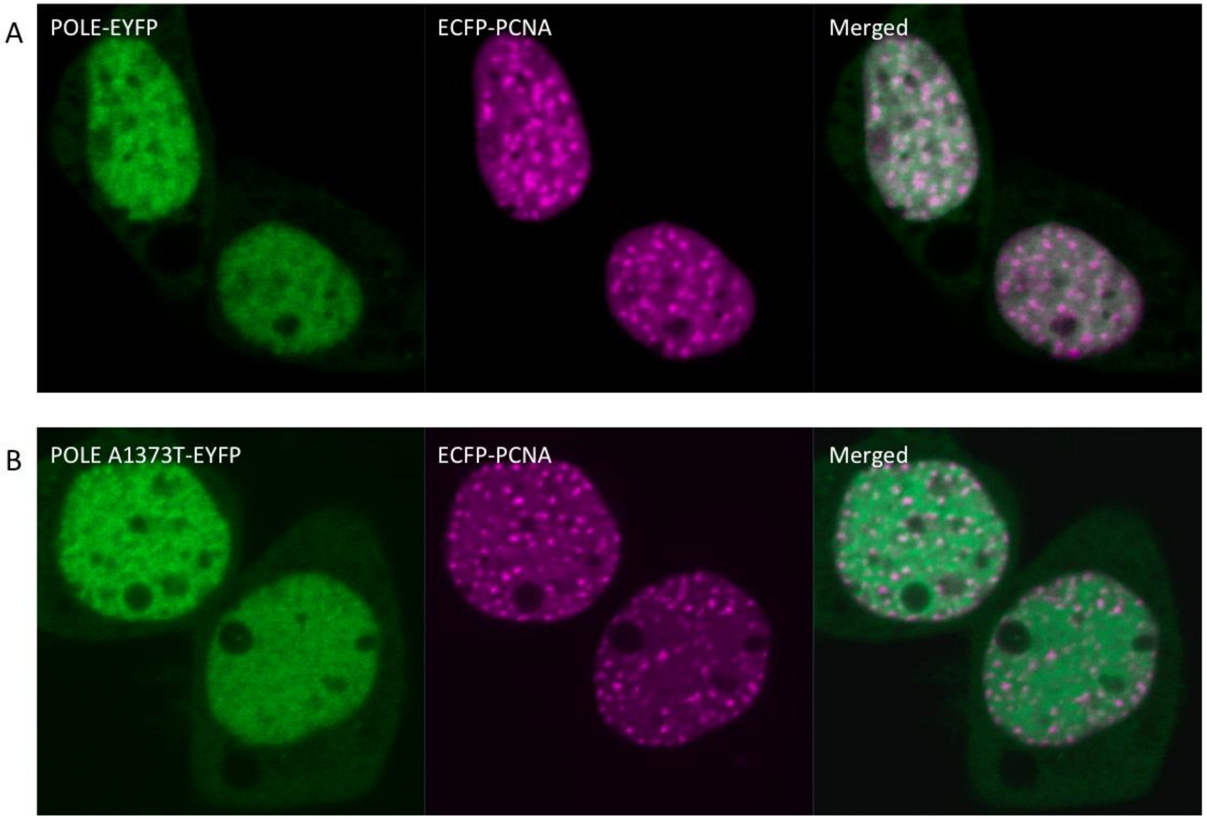

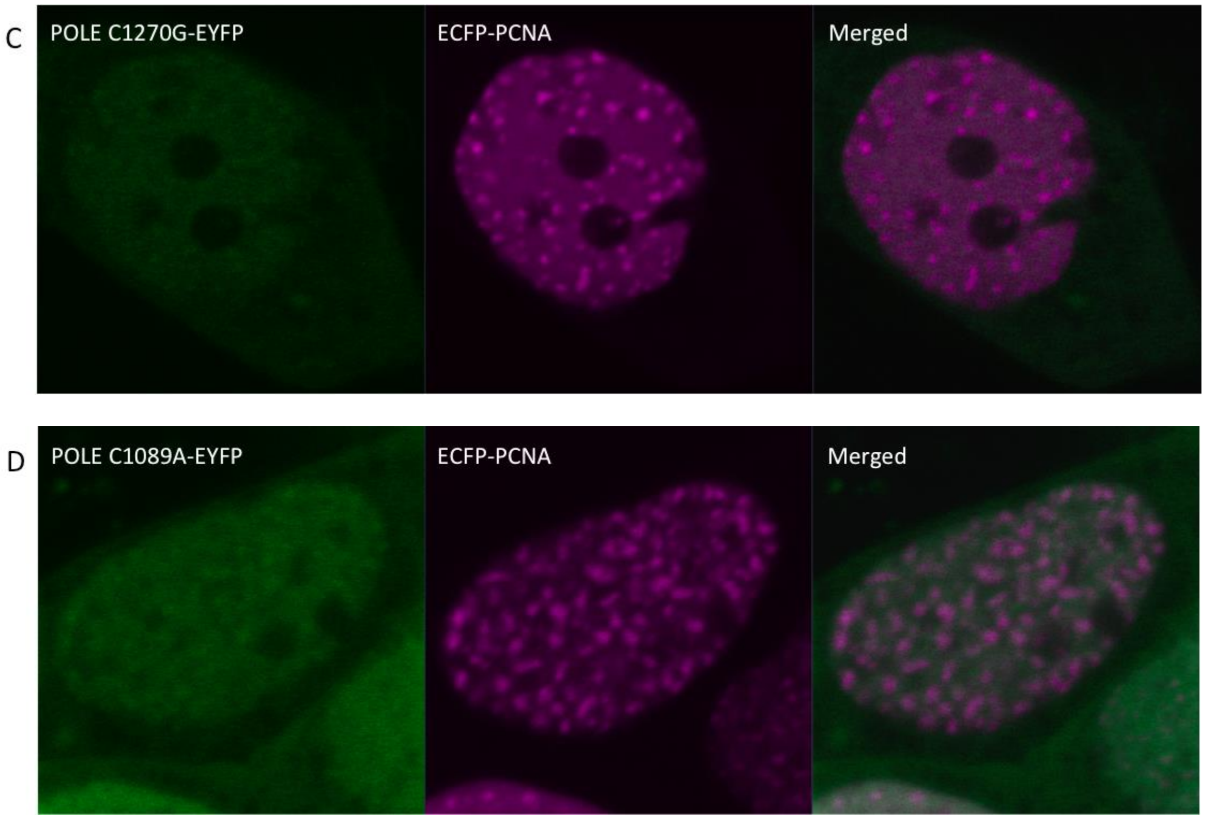

Fig. S3-1 Laser scanning confocal microscopy time course study of POLE-EYFP fusion protein. HEK293T cells were transfected with pEF6-POLE-EYFP and ECFP-PCNA plasmid. Images were taken 13 hours after transfection. (A) POLE-WT, (B) POLE c.1373A>T (p.Tyr458Phe), (C) POLE c.1270C>G (p.Leu424Val), and (D) POLE c.1089C>A (p.Asn363Leu).

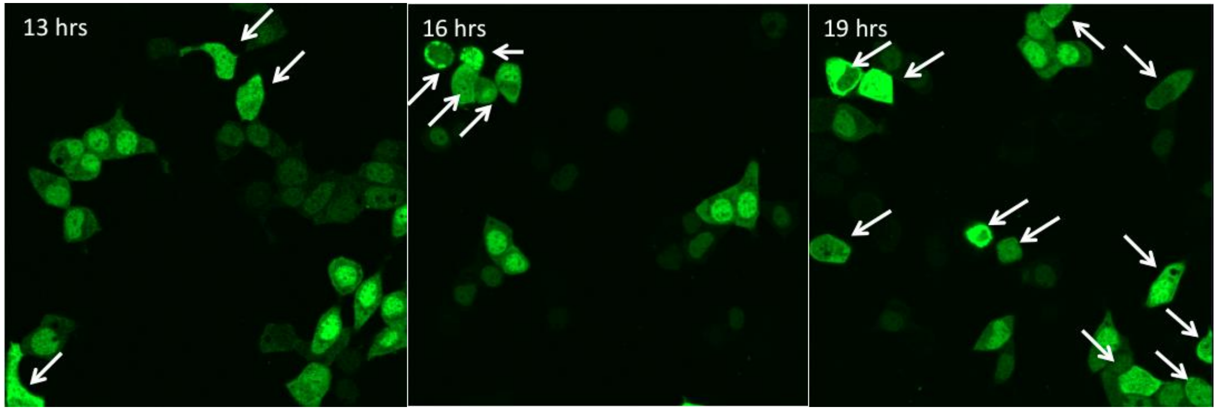

.

Fig. S3-2 Laser scanning confocal microscopy time course study of POLE-EYFP fusion protein. HEK293T cells were transfected with pEF6-POLE-EYFP Images were taken 13, 16, and 19 hours after transfection. POLE-EYFP localization to the cytosplasm, relative to nuclear localization, increases over time as indicated by arrows

Table S1. Oligonucleotide sequences used for gene editing of *POLE* c.1373A>T by CRISPR/Cas9. All sequences are written 5'->3'. The single stranded oligonucleotide used as a repair template includes two 90-nt homology arms (lower case letters), silent mutations (in red), and the mutation of interest (red, highlighted).

Description	Sequence
SpCas9:sgRNA vector	GAGGGCCTATTTCCCATGATTCC
sgRNA	top: CACCGAGCATCTGACACAGAATACG<br>bottom: AAACCGTATTCTGTGTCAGATGCTC
Single stranded oligonucleotide repair template	aggccaggctttgctttctgtgcttcacacttgaccctgggctcttgatttttgatggccctgctctctggcgttctct cctcagactctAGCGACCTTttctgtgtcagatgctgtcgccacttactacctgtacatgaagtacgtccac ccattcatctttgctctgtgcaccattattcccatgga
POLE c.1373 primers	Forward: TATGATCCCGTGGAGCTAGA<br>Reverse: CAGGCAAAGTCCAGGAACCTA

Table S2. Primers used for site-directed mutagenesis

Name	Primers (5′-3′)
POLE c.1089C>G (p.Asn363Lys)	Forward:<br>CCAGTCAAAGAAGTCACCCTTGTATGTAACTATTACTGAAGG<br>Reverse:<br>CCTTCAGTAATAGTTACATACAAGGGTGACTTCTTTGACTGG
POLE c.1373A>T (p.Tyr458Phe)	Forward:<br>CAACAGCATCTGAAACTGAAAATTGAGCAAGAACTTGAGGTT<br>Reverse :<br>AACCTCAAGTTCTTGCTCAATTTTCAGTTTCAGATGCTGTTG
